# Application of Light-Emitting Diodes for Improving the Nutritional Quality and Bioactive Compound Levels of Some Crops and Medicinal Plants

**DOI:** 10.3390/molecules26051477

**Published:** 2021-03-09

**Authors:** Woo-Suk Jung, Ill-Min Chung, Myeong Ha Hwang, Seung-Hyun Kim, Chang Yeon Yu, Bimal Kumar Ghimire

**Affiliations:** 1 Department of Crop Science, College of Sanghuh Life Science, Konkuk University, Seoul 05029, Korea; jungw@konkuk.ac.kr (W.-S.J.); imcim@konkuk.ac.kr (I.-M.C.); kshkim@konkuk.ac.kr (S.-H.K.); 2 Interdisciplinary Program in Smart Science, Kangwon National University, Chuncheon 200-701, Korea; mwung.ha5471@daum.net (M.H.H.); cyyu@kangwon.ac.kr (C.Y.Y.)

**Keywords:** light emitting diodes, anthocyanins, antioxidant enzymes, organogenesis, phenolic acid, carotenoid, tocopherol

## Abstract

Light is a key factor that affects phytochemical synthesis and accumulation in plants. Due to limitations of the environment or cultivated land, there is an urgent need to develop indoor cultivation systems to obtain higher yields with increased phytochemical concentrations using convenient light sources. Light-emitting diodes (LEDs) have several advantages, including consumption of lesser power, longer half-life, higher efficacy, and wider variation in the spectral wavelength than traditional light sources; therefore, these devices are preferred for in vitro culture and indoor plant growth. Moreover, LED irradiation of seedlings enhances plant biomass, nutrient and secondary metabolite levels, and antioxidant properties. Specifically, red and blue LED irradiation exerts strong effects on photosynthesis, stomatal functioning, phototropism, photomorphogenesis, and photosynthetic pigment levels. Additionally, ex vitro plantlet development and acclimatization can be enhanced by regulating the spectral properties of LEDs. Applying an appropriate LED spectral wavelength significantly increases antioxidant enzyme activity in plants, thereby enhancing the cell defense system and providing protection from oxidative damage. Since different plant species respond differently to lighting in the cultivation environment, it is necessary to evaluate specific wavebands before large-scale LED application for controlled in vitro plant growth. This review focuses on the most recent advances and applications of LEDs for in vitro culture organogenesis. The mechanisms underlying the production of different phytochemicals, including phenolics, flavonoids, carotenoids, anthocyanins, and antioxidant enzymes, have also been discussed.

## 1. Introduction

Among the different environmental factors, light is the most important factor that affects plant gene expression, enzyme activity, growth, development, and nutritional composition [1,2,3]. Recent studies have reported the effects of light quality (spectral specificity) on phytochemical accumulation in plants [4,5,6,7,8,9]. Several studies on light-emitting diodes (LEDs) have reported improvement in the nutritional quality of plants grown under LED irradiation. For example, increased accumulation of primary secondary metabolites, starch, simple sugars, proteins, vitamin C, and phenolic compounds including anthocyanins has been observed in plants grown under LED irradiation [10,11,12]. Recently, LEDs have been increasingly applied in agro-farming and in vitro culture owing to their advantages over conventional light sources. For example, LEDs are more energy-efficient, have a longer life span, and exhibit higher spectral specificity than standard lamps (fluorescent [FL] lamps) [13]. Moreover, the application of monochromatic light is important in research centers [14]. FL light encompasses a wide range of wavelengths (350–750 nm) and is suitable as a light source for several plant species; however, it has certain disadvantages, including higher electricity consumption, more heat emission, and greater variation in radiation wavelengths than LEDs [15]. Comparatively, LEDs demonstrate lower heat emission and higher energy conversion efficiency than other conventional artificial light sources [13,16]. Another desirable characteristic of LEDs over other light systems is that they can be positioned close to plants and controlled to emit specific wavelengths [13]. Thus, the beneficial aspects of LEDs over FL lamps have recently led to their wide application in the field of agriculture for post-harvest uses, preservation, disease resistance, and development of in vitro culture systems [17].

Light is a primary factor that affects plant development, physiology, and cellular differentiation [18]. Environmental factors, including light spectrum types, are important signaling components of plant physiology and metabolite synthesis [19,20]. Photoreceptors present in plants and matching spectral attributes are the main factors that regulate plant morphogenesis and metabolite synthesis [21]. The application of artificial light in different plant species has been investigated in several previous studies to determine its effects on the stimulation of plant metabolite production and photosynthesis [22]. LED irradiation has been found to be effective in stimulating plant metabolite production after harvest and during development [23]. Previous studies have demonstrated the effective use of LEDs in in vitro growth and organogenesis of plants, including banana [24], strawberry [25], chrysanthemum [26], and potato [27].

Plants perceive light through photoreceptors, such as red light-sensitive phytochromes, blue light-sensitive phototropins, and cryptochromes, which regulate several specific physiological responses, including organogenesis and metabolite synthesis [28,29]. The success of in vitro plant regeneration and metabolite synthesis relies greatly on the spectral quality of light and photon efficiency of the light source [21]. According to Samuolienė et al. [5], a considerable challenge associated with tissue culture is to provide high quality light of controlled intensities in sufficient quantity for plant development. Numerous studies have reported the successful application of LEDs in in vitro shoot organogenesis and plant growth. For example, significantly improved biomass yield, increased shoot regeneration, and improved adaptability and survival rate of regenerated plants have been reported [30,31,32,33]. Improved secondary metabolite accumulation and in vitro root growth have been reported by Xu et al. [34] in *Cunninghamia lanceolata* and Nadeem et al. [35] in *Ocimum basilicum* under LED irradiation. Plant growth, development, and metabolite production are strongly affected by the light spectrum of the LEDs. A previous study suggested that the blue light spectrum was involved in morphogenesis, phototropism, the leaf photosynthetic process, and stomatal opening [36]. Red LEDs emit a spectrum very close to the maximum absorbance for both chlorophyll and phytochromes. The effects of light spectra on plant physiology vary among species, thereby causing significant variation in biomass yield and plant production.

The present review highlights the variation in in vitro organogenesis and somatic embryogenesis among different plant species grown under LED irradiation. Additionally, this review elucidates the effects of LED irradiation on secondary metabolite accumulation and antioxidant properties of plants. Finally, the effects of LED irradiation on the expression of genes related to the production of phenolic acids, flavonoids, carotenoids, and chlorophyll have been discussed.

## 2. Results

### 2.1. Effects of LED Irradiation on Antioxidant Enzymes

Light is an important factor that affects several biochemical pathways in plants during their growth and development. Antioxidant compounds, such as phenolic acids, vitamins, anthocyanins, carotenoids, and α-tocopherol, are widely affected by the duration of light exposure and spectral wavelength of light sources [37,38,39]. Spectral quality affects the antioxidant enzyme activity and antioxidant properties of plants [40]. A previous study reported the importance of light in antioxidant enzyme metabolism [41]. For instance, the combination of red and blue light at a ratio of 1:1 enhanced the activity of antioxidant enzymes, including catalase (CAT), peroxidase (POD), superoxide dismutase (SOD), and ascorbate peroxidase (APX), in *Carpesium triste* Maxim [42]. Increase in the activity of antioxidant enzymes, such as CAT, was found to be related to the delay in the onset of leaf senescence in *C. triste* [42], wheat [43], pea [44], and banana [45] (Table 1). Numerous previous studies have shown the differential responses of antioxidant enzymes in plants grown in vitro under different light conditions. For instance, callus cultures of *Cynoglossum officinale* grown under dark conditions showed increased CAT activity, whereas those grown under blue and white light conditions showed reduced CAT activity [46]. According to Causin et al. [43], blue light plays an important role in preventing cell senescence and decreasing cellular oxidative damage by enhancing CAT activity in wheat plants. In another study, blue light strongly activated catalase isozyme 1 (CAT-1) in rye plants [47]. CAT-1 is known to eliminate photorespiratory H_2_O_2_ [47], indicating its positive association with plant antioxidant defense mechanisms. Differential effects of LED irradiation on in vitro shoot organogenesis and antioxidant enzyme activities and variations in reactive oxygen species (ROS) levels have been reported in different plant species [48]. A significant decrease in SOD activity has been observed during the initial stage of organogenesis in *Curculigo orchioides* grown under combined red and blue LED irradiation [49]. The highest SOD activity was observed after two weeks of red LED irradiation. Moreover, Franck et al. [50] demonstrated a close association between shoot bud formation and enhanced SOD activity during in vitro organogenesis of *Prunus avium* and strawberries grown under blue LED irradiation. In similar studies, enhanced SOD and CAT activities were observed during adventitious shoot formation in *Gladiolus hybridus* [51] and *Albizia adorratissima* [52] grown under blue LED irradiation. It has been reported that CAT plays an important role in shoot organogenesis, and enhanced CAT activity is associated with increased adventitious shoot formation in plants [52]. High CAT activity during shoot initiation is associated with H_2_O_2_ dismutation [53]. Additionally, Causin et al. [43] observed increased CAT activity and reduced cell senescence in wheat plants exposed to blue light.

### 2.2. Effects of LED Irradiation on In Vitro Organogenesis

LEDs have drawn considerable attention as suitable alternative light sources for the in vitro propagation, mass propagation, shoot regeneration, and root culture of various plant species. Due to technological advancement and flexibility of LED spectral wavelength, LEDs have been successfully applied for in vitro organogenesis of plant species (Table 2). Several studies have reported the effects of LED irradiation on the carbohydrate metabolism and micropropagation of plant species [101,102,103]. Many previous studies have demonstrated the varying in vitro shoot and root organogenesis-promoting effects of irradiation with LED combinations, depending on the various plant parts and species [104,105,106]. Blue and red LED irradiation was found to stimulate shoot organogenesis in potato [104] and vanilla [107] and enhance bulbet organogenesis in *Lilium* [108]. Increased shoot regeneration was observed in *A. distichum* irradiated with combined red and blue LEDs [109]. Other studies demonstrated the stimulatory role of monochromatic blue or red LED irradiation in shoot organogenesis [1,110,111]. Increased shoot elongation was observed in *Oncidium* [112], and blueberries [113,114] irradiated with red LED. In a similar study, shoot elongation was increased in sugarcane irradiated with blue and red LED combinations [110].

In several studies, LED irradiation of in vitro plants increased their biomass. For example, irradiation with blue and red LED combinations resulted in enhanced biomass during the in vitro culture of *Achillea millefolium* [115], Densribium [1], blueberries [114], sugarcane [110,116], and chrysanthemum [26]. In addition to biomass, chlorophyll content was increased in different plant species cultured under LED irradiation [24,42,117,118,119]. In similar studies, increased total carotenoid level was reported in shoot cultures irradiated with different LEDs [119,120,121]. Tuan et al. [120] observed elevated expression of carotenoid biosynthesis-associated *PSY, ZDS, CHXB*, and *ZEP* genes. LED irradiation of various cultured plants also increased in vitro adventitious root induction. For example, the adventitious root-promoting effects of LED irradiation were observed in strawberries [122], *chrysanthemum* [123], chestnuts [124], *Oncidium* [113], and *C. lanceolata* [34]. The effects of spectral differences in light quality on somatic embryo formation have been reported in *Peucedanum japonium* [125], *Coffea canephora* [126], *Pinus densiflora* [127], *Pinus taeda*, and *Pinus elliottii* [128]. However, somatic embryo formation and germination were observed on irradiation with different combinations and quantities of LEDs. Jung et al. [129] observed an increase in the polyphenol content of rice seedlings grown in vitro under irradiation with different LED combinations.

In several instances, increased bioactive compound production observed in plant species grown in vitro could be maintained under irradiation with different LEDs [35,46,130,131,132,133,134]. Additionally, increased total phenol and total flavonoid contents were observed in different plants irradiated with different LEDs [1,35,42,119,135]. Recently, increased phytochemical levels have been recorded in important crops and medicinal plants. For example, ascorbic and dehydroascorbic acids were observed in *Lycopersicon esculentum* cv. ‘House Momotaro’ & ‘Mini Carol’ [136], and myrcene and limonene were observed in *Lippia rotundifolia* Cham maintained under blue LED irradiation [137]. Irradiation with red and blue LED combinations enhanced phytochemical levels in *Bacopa monnieri* L. [138] and *Plectranthus amboinicus* (Lour.) Spreng [139]. Increased antioxidant activity was significantly correlated with enhanced phytochemical concentration in LED-irradiated plants. Moreover, a substantial increase in antioxidant enzymatic activity was observed in plants grown in vitro under irradiation with various LEDs. For instance, Gupta and Sahoo [80] observed an increase in APX activity in *C. orchioides* cultured under red LED irradiation. Similarly, enhanced POD activity was observed in *C. orchioides* irradiated with blue LEDs [80]. Additionally, changes in the antioxidant enzyme activity and polyphenol concentration were observed in LED-irradiated plants cultured in vitro [106,140,141], indicating a close association between LED irradiation and plant phytochemical composition and antioxidant activities.

**Table 2:** Effect of light emitting diodes on in vitro plant propagation.

Plant Species	Type of LED	Metabolites/Enzyme/Gene	Biological Activity	References
*Lippia gracilis* Schauer	Blue-LED	Total chlorophyll, total carotenoid, carvacrol, *E*-caryophyllene	Bioactive compound production	Lazzarini et al. [117]
*Brachypodium distachyon* (L.)	Red:Blue:White LED	*PAL, F5H* Superoxide dismutase, Catalase	Gene expression, antioxidant enzyme expression	Mamedes-Rodrigues et al. [141]
*Hyptis marrubioides* Epling	White and Blue-LED	Rutin	Bioactive compound production	Pedroso et al. [130]
*Cunninghamia lanceolata*	Red:Blue:Purple:Green (8:1:1:1) LED	Peroxidase, catalase	Root growing, antioxidant enzyme expression	Xu et al. [34]
*Ocimum basilicum* L.	Red, Blue, White LED	Total flavonoid, peonidin, cyaniding (Red LED), rosmarinic acid, eugenol (Blue LED), chicoric acid (White)	Bioactive compound production	Nadeem et al. [35]
*Lycopersicon esculentum* cv. ‘House Momotaro’ & ‘Mini Carol’	Blue LED	Ascorbic acid, dehydroascorbic acid	Antioxidation	Zushi et al. [136]
*Boehmeria nivea* cv. ‘Zhongsizhu 1′	Red and Orange LED	Total chlorophyll (Red), malondialdehyde (+), superoxide dismutase, peroxidase (Orange)	Bioactive compound production, antioxidant enzyme expression	Rehman et al. [118]
*Schisandra chinensis* (Turcz.)	Blue LED	Chlorogenic acid, gallic acid, protocatechuic acid	Biomass increase, Bioactive compound production	Szopa and Ekiert [131]
*Bacopa monnieri* L.	Red and Blue LED	Triterpenoid saponin glycosides	Bioactive compound production	Watcharatanon et al. [138]
*Scutellaria baicalensis* Georgi	Blue LED	Total carotenoid, *PSY*, *ZDS*, *CHXB*, *ZEP*	Bioactive compound production, gene expression	Tuan et al. [120]
*Cnidium officinale* Makino	Red and Blue (1:1) LED	Total phenol, total flavonoid, ascorbate peroxidase	Bioactive compound production, antioxidant enzyme exprssion	Adil et al. [46]
Grapes	Blue LED	Chlorophyll	photosynthetic compound	Poudel et al. [110]
*Canavalia ensiformis*	Red and Blue (1:3) LED	Total phenol, total chlorophyll, total carotenoid	Biomass increase, callus induction, bioactive compound production, antioxidation	Saldarriaga et al. [119]
*Rhodiola imbricata* Edgew	Blue LED	Salidroside, total phenol, total flavonoid	Bioactive compound production	Kapoor et al. [135]
*Lepidium sativum* L.	White, Blue, Green LED	Total phenol (White), *p*-coumaric acid (Blue), superoxide dismutase, peroxidase (Green)	Bioactive compound production, antioxidant enzyme exprssion	Ullah et al. [132]
*Solanum tuberosum* cv. ‘Zhuanxinwu’	Blue LED	Anthocyanin	Bioactive compound production	Xu et al. [142]
*Ajuga bracteosa*	Blue LED	Total phenol, total flavonoid	Bioactive compound production	Rukh et al. [133]
*Vitis vinifera* cv. “Manicure Finger”	Blue LED	Total chlorophyll, total carotenoid	Bioactive compound production	Li et al. [143]
*Lippia rotundifolia* Cham	Blue LED	Myrcene, limonene	Bioactive compound production	De Hsie et al. [137]
*Pfaffia glomerata* accessions (Ac22, Ac43)	Red and Blue (1:1) LED	Anthocyanin, 20-hydroxyecdysone, peroxidases, catalase	Bioactive compound production, antioxidant enzyme exprssion	Silva et al. [140]
*Lippia filifolia* Mart. & Schauer	Red, Blue LED	Malondialdehyde (-)	Bioactive compound production	Chaves et al. [134]
*Drosera burmannii* Vahl, *Drosera indica* L.	Blue LED	Plumbagin	Bioactive compound production	Boonsnongcheep et al. [144]
Potato	Red and Blue LED	-	Shoot elongation	Edesi et al. [104]
Lilium	Red and Blue LED	-	Bulbet organogenesis	Lian et al. [108]
Vanilla	Red and Blue LED	-	Shoot organogenesis	Bello-Bello et al. [107]
*A. distichum*	Red and Blue LED	-	Shoot regeneration	Lee et al. [109]
*R. glutinosa,*	Red LED	-	Shoot elongation	Hahn et al. [22]
Sugarcan	Blue and Red LED	-	Shoot elongation	Silva et al. [139]
*A. milletolium*	Blue and Red LED	-	Enhanced biomass	Alvarenga et al. [115]
*Densribium*	Blue and Red LED	-	Enhanced biomass	Lin et al. [1]
Blue-berry	Blue and Red LED	-	Enhanced biomass	Hung et al. [114]
*Crysanthemum*	Blue and Red LED	-	Enhanced biomass	Kim et al. [26]
Sugarcan	Blue and Red LED	-	Enhanced biomass	Maluta et al. [116]
*Castanea crenata*	Red-LED	-	Shoot elongation	Park and Kim et al. [145]
*Oncidium*	Red-LED	-	Shoot elongation	Chung et al. [113]
Blue berry	Red LED	-	Shoot elongation	Hung et al. [112,114]
Banana	Red-LED	-	Chlorophyll	Do Nascimento Vieira et al. [24]
*C. orchioides*	Red-LED, Blue-LED	APX, POX	Enzyme activity	Dutta G. and Sahoo [80]

### 2.3. Effects of LED Irradiation on Tocopherol Biosynthesis in Crops

Tocopherols, synthesized through the isopropenoid pathway, are associated with the antioxidant properties of green plants [146]. These phytochemicals play a key role in protecting the photosynthetic membranes and apparatus from high-intensity light stress [147]. A previous study showed direct interaction between photoreceptor activation and tocopherol content in plants [148]. A significant increase in ɣ-tocopherol content and the suppression of α-tocopherol content were reported in barley irradiated with red LEDs [81]. Moreover, yellow LED irradiation effectively enhanced tocopherol accumulation in apples [82], demonstrating the species-dependent effects of LED irradiation. Similar results were reported in basil, whereas combined irradiation with blue and red LEDs, compared with only blue LED irradiation, enhanced α-tocopherol content in parsley [53]. Koga et al. [81] proposed that the suppression of homogentisate phytyltransferase, an enzyme that regulates the total tocopherol content in plants, might lower the tocopherol concentration in blue LED-irradiated sprouts. Moreover, in another study, irradiation with blue LEDs at a lower dosage resulted in an increase in the total tocopherol content in beets [83]. Thus, it is possible that LED irradiation can interact with the enzymes involved in tocopherol biosynthesis pathways. However, in another study, irradiation with HPS lamps combined with red LEDs significantly enhanced α-tocopherol accumulation in parsley extracts [83], indicating that red or blue LED irradiation is solely insufficient to regulate tocopherol biosynthesis in plants.

### 2.4. Effects of LED Irradiation on Carotenoid Biosynthesis in Crops

Carotenoids, including β-carotene and lutein, are present in most green plants and green algae and are associated with light harvesting and the transfer of energy to the reaction center of photosystems [149,150]. They also deactivate ROS formed under extreme light stress to protect the photosynthetic apparatus [151]. Moreover, carotenoid consumption has been linked to several health benefits in humans, including heart disease and cancer prevention, and it has been reported to be closely associated with ophthalmic health [152,153,154,155]. Many previous studies have reported the effects of both spectral quality and light intensity on carotenoid biosynthesis in plants. Among the different LEDs, red LED irradiation enhanced β-carotene accumulation in pea plants [84]. Other studies reported that red LED irradiation resulted in an increase in β-cryptoxanthin content in citrus fruits [156] and lycopene content in tomatoes [157]. The duration of LED irradiation affected total carotenoid content and carotenoid biosynthesis in the growing plants. Compared to combined red and blue LED irradiation, blue LED irradiation for a short duration significantly increased β-carotene and violaxanthin accumulation in broccoli microgreens [89]. In a similar study, BC levels in pea plants were increased after blue LED irradiation for a short duration [84]. However, β-carotene and lutein levels in buckwheat sprouts were decreased following blue LED irradiation, compared with white LED irradiation [120]. As shown in Table 1, the LED source markedly affected carotenoid accumulation and was significantly associated with gene expression during carotenoid biosynthesis in the plants. The variation in carotenoid content in the plants irradiated with LEDs could be attributed to the differential expression of genes associated with carotenoid synthesis. For instance, buckwheat sprouts grown under irradiation with different LEDs showed increased expression of *FtPSY, FtLCYB, FtCHXB, FtCHXE, FtLCYe,* and *FtZEP* genes, which are associated with carotenoid biosynthesis, following white LED irradiation [120]. In a similar study, Zhang et al. [88] observed the upregulation of the expression of carotenoid biosynthesis-associated genes, such as *CitPSY, CitZDS, CitPDS,* and *CitLCY,* in citrus species, indicating a differential stimulatory role of LED irradiation in the regulatory mechanism of carotenoid biosynthesis in plants.

### 2.5. Effects of LED Irradiation on Flavonoid Biosynthesis in Crops

Flavonoids are widely distributed phytochemicals found in plants and are involved in multiple mechanisms, including protection against pathogens and ultraviolet (UV) radiation, flower coloration, and male fertility [158,159,160]. Additionally, these phytochemical compounds are involved in plant coloration, protection of leaf cells from photooxidative damage [161], stress response, and other physiological activities [162,163]. Light is an important abiotic factor that affects flavonoid accumulation and flavonoid biosynthesis-related gene expression in plant species [164]. Numerous previous studies have reported the key role of LEDs in flavonoid biosynthesis in plants. Blue LED irradiation increased anthocyanin concentration in grapes [90]. Upregulation of the expression of VIMYBA1-2, VIMYBA2, and VvUFGT genes, which are associated with anthocyanin biosynthesis, was also observed. Similarly, Thwe et al. [91] observed increased anthocyanin accumulation in buckwheat grown under blue LED irradiation and wide variation in FtPAL, FtANS, and FtDFR expression in buckwheat sprouts. In another study, a positive correlation between anthocyanin accumulation and flavonoid synthesis-related gene expression, including that of 4-coumaryol CoA-ligase (4CL) and phenylalanine ammonia synthase, was observed in *Cyclocarya paliurus* grown under blue LED irradiation [57]. Park et al. [72] reported an increase in the levels of rosmaric acid, tilianin, and expression of genes encoding phenylpropanoid biosynthesis-related enzymes, such as cinnamate 4-hydroxylase (C4H), chalcone isomerase (CHI), and RAS, in Acaulospora rugosa under white LED irradiation, compared with irradiation with other LEDs. Similarly, enhanced gallic acid and quercetin accumulation and decreased *p*-coumaric acid and epicatechin levels were observed in wheat sprouts grown under blue LED irradiation [49]. Irradiation with a combination of blue and red LEDs at a ratio of 1:4 resulted in an increased expression of genes encoding flavonoid synthesis-related enzymes, such as phenylalanine ammonia lyase (PAL), chalcone synthase (CHS), CHI, and flavonol synthase, in *Anoectochilus roxburghii*, further resulting in increased flavonoid accumulation [60].

### 2.6. Effects of LED Irradiation on Anthocyanin Biosynthesis in Crops

Anthocyanins are soluble flavonoids that are widely distributed in plants and are associated with seed dispersal, pollination, stress resistance, and flower coloration. They are widely used in the food industry for coloring purposes [165]. Additionally, these phytochemicals are known for their antioxidant properties, including protection of the photosynthetic apparatus and DNA from harmful radiation and cold stress, and they play key roles in drought resistance [166,167]. Previous studies have reported the effects of LED irradiation on anthocyanin accumulation in plants [58]. Irradiation with a red and far-red LED combination has been reported to increase total anthocyanin (TA) content in lettuce plants [92,93]. However, irradiation with a deep red LED alone reduced total anthocyanin content in mustard plants [94]. TA concentration was significantly increased in other vegetables, such as cabbage [95] and Chinese kale sprouts [168], following red LED irradiation. Moreover, irradiation with a combination of a red LED with HPS lamp resulted in higher TA accumulation in green vegetables than irradiation with red LEDs alone [169]. Green vegetables, such as lettuce and romaine baby leaves, have been reported to show higher TA content when grown under green LED irradiation than when grown under red LED irradiation [6,40]. Moreover, TA accumulation was higher in *Camellia sinensis* (L.) O. Kuntze ‘Zhonghuang 3′ grown under blue LED irradiation [62]. These results indicated that TA biosynthesis in plants depended on not only the light wavelength but also the plant species. Anthocyanin concentration was increased in apples grown under red LED irradiation [96]. According to this report, red LED irradiation upregulated the expression of *MD-MYB10* and *MdUFGT* genes, which are related to anthocyanin biosynthesis [96]. Moreover, irradiation of grapes with both blue and red LEDs upregulated the expression of anthocyanin biosynthesis-related genes, such as MYB transcription factor genes [97]. In another study, the expression of anthocyanin synthesis-related genes, including *V1MYBA1-2*, *VIMYBA2*, and *VvUFGT*, was increased with the enhancement in anthocyanin accumulation in grape berries irradiated with blue LEDs [90].

### 2.7. Effects of LED Irradiation on Phenolic Acid Biosynthesis in Crops

Phenolic compounds are ubiquitous in most higher plants and are associated with plant defense systems against abiotic and biotic factors, including UV radiation, high temperature, excess light, pathogen attack, and wounding [170,171]. Phenolic compounds are formed via the shikimate pathway in plants. Phenylalanine, an intermediate compound formed in these pathways, is converted into phenolic compounds by PAL, which is widely regulated by light-responsive factors and ROS formed under excess light [142,172]. Some studies have reported the effects of LEDs on phenolic acid accumulation in plants. Among the different LEDs, irradiation with red LED was effective in increasing the total phenolic content (TPC) in basil [53]. A stimulatory effect of red LED irradiation on TPC in various vegetables, including radish, wheat, and lentil, was observed. Moreover, a positive effect of irradiation with red LEDs, combined with other LEDs on TPC in basil microgreens was observed. However, red LED irradiation exerted a negative effect on TPC in parsley microgreens [53]. In contrast, Qian et al. [95] and Brazaityte et al. [169] found that red LED irradiation did not affect TPC in Chinese kale sprouts and *Brassica* microgreens, respectively. A similar trend was also observed in lettuce leaves [40,173]. Blue LED irradiation resulted in an increase in TPC in growing Chinese kale sprouts [95]. Other studies have reported increased TPC in Chinese cabbage and lettuce irradiated with blue LEDs alone compared with those irradiated with red LEDs alone or a combination of red and blue LEDs [98], indicating that the effects of LEDs on TPC varied among plant species. Several studies have investigated the levels of phenolic compounds in plant species grown under LED irradiation [55,57,58,68,72,174,175]. Chung et al. [4] reported an increase in malonyldaidzin, malonyl genistin, salicylic acid, *p*-hydrobenzoic acid, and gentisic acid levels in *Pachyrhizus erosus* grown under red LED irradiation. An increased concentration of *p*-coumaric acid was observed in *P. erosus* grown under blue LED irradiation. The accumulation of phenolic compounds, such as *p*-coumaric, gallic, ferulic, and hydroxybenzoic acids, was increased in wheat sprouts irradiated with blue LEDs [99]. Several studies have shown an increase in antioxidant activity and phytochemical accumulation in plants irradiated with different LEDs [4,175,176].

Irradiation with blue LEDs triggered increased phenolic compound accumulation and phenolic compound biosynthesis-related gene expression [59,74,77]. Irradiation with red LEDs alone was also shown to enhance the concentrations of important phytochemicals in various plant species [65,69,70,73]. Additionally, irradiation with LED combinations at different ratios enhanced phytochemical contents in plant species [54,56,67,71,76,177,178,179]. The increase in phenolic compound content was positively correlated with the expression of *TaPA1, TAPA2, TaC4H, TaCHI, TaCHS,* and *TaF3H* genes; these genes are involved in phenolic compound synthesis through the phenylpropanoid biosynthesis pathway [65]. The highest levels of select flavonoids (kaempferol, isoquercitrin, and quercetin) and enhanced relative expression of genes encoding key enzymes, such as PAL, 4CL, and CHS, were observed in *Cyclocarya paliurus* irradiated with blue LEDs [100].

## 3. Conclusions and Future Prospects

In this review, we aimed to provide updates on the innovative use of LEDs in improving nutritional quality of plants grown in vitro and in vivo. Moreover, in the present review, we summarized the expression patterns of various genes related to phytochemical biosynthesis in response to different LED spectral wavelengths. It is important to identify the appropriate light quality and intensity to increase the quantity and quality of important phytochemicals associated with nutrition and human health. It can be concluded from this overview of research that the flexibility of LED irradiation allows the enhancement of nutritional levels of vegetables and phytochemical contents of plant species. Moreover, irradiation with LED combinations at different ratios and combination of LEDs with normal light (FL) sources can enhance phytochemical content, biomass, and nutritional quality of vegetables and medicinal plants. However, detailed studies on the association between LEDs and their phytochemical accumulation-promoting effects as well as the underlying physiological and molecular mechanisms are required. We observed that different plant species respond differentially to various LED spectral wavelengths. Therefore, further research is required to understand the application of LEDs for the successful growth and mass propagation of plants.

## Figures and Tables

**Table 1:** Effect of light emitting diodes on secondary metabolites compositions and biological activity.

Plant Species	Type of LED	Secondary Metabolites/ Enzyme/Gene	Biological Activity	References
*Lactuca sativa* var. *crispa* “Green Oak Leaf”	Blue LED	Carotenoid and chlorophylls	Bioactive compound production	Chen et al. [8]
*Lactuca sativa* L. cv. Butterhead	Red, Blue and Green (4:1:1) LED	*LHCb, PsbA*	Gene expression	Bian et al. [54]
*Perilla frutescens* var. crispa	Red LED	Rosmarinic acid, caffeic acid	Bioactive compound production, biomass increase	Nguyen and Oh [55]
*Lactuca sativa* L. cv. Banchu Red Fire	BlueLED	Polyphenol and carotenoid	Bioactive compound production,	Johkan et al. [56]
*Pisum sativum* L.	Blue LED	Chlorogenic acid	Antioxidation	Liu et al. [57]
*Lactuca sativa* L. var Lollo rosso	Red and Blue(1:5) LED	Chlorogenic acid	Bioactive compound production	Azad et al. [58]
*Abelmoschus esculentus* L.	Blue LED	*PAL*	Gene expression	Wilawan et al. [59]
*Brassica alboglabra* Bailey. cv. Lvbao	Red and Blue (2:1) LED	Amino acids	Bioactive compound production	Zhang et al. [56]
*Pachyrhizus erosus* L.	Blue LED	l-phenylalanine	Antioxidation	Chung et al. [4]
*Anoectochilus roxburghii*	Red and Blue(8:2) LED	*PAL, CHS, CHI, and FLS,*	Gene expression	Gam et al. [60]
*Brassica juncea*	Blue LED	4-hydroxybenzoic acid	Bioactive compound production	Park et al. [61]
*Camellia sinensis* (L.) O. Kuntze ‘Zhonghuang 3′	Blue LED	Anthocyanins, catechins, *CRY2/3, SPA, HY5*	Bioactive compound production, Gene expression	Zheng et al. [62]
*Coriandrum sativum* L.	Red, Blue and Far red (81.5:12.5:6) LED	Ascorbic acid	Biomass increase, Bioactive compound production	Nguyen et al. [55]
*Stevia rebaudiana*	Red:Far red: Blue (5:6.1), LED	*UGT85C2*	Gene expression	Yoneda et al. [63]
*Perovskia atriplicifolia*	Blue LED	δ-3-Carene	Bioactive compound production	Ghaffari et al. [64]
*Momordica charantia*	Red LED	Charantin, *AACT, MVD, IDI,* *FPS1, FPS2, CAS2*	Bioactive compound production, gene expression	Cuong et al. [65]
*Brassica napus* sprouts	Blue LED	Caffeic acid	Bioactive compound production	Park et al. [66]
*Lactuca sativa* L.	Red and Blue (1:3) LED	Ascorbate, *GMP, GME,* *GGP, GGP, GLDH*	Bioactive compound production, gene expression	Zha et al. [67]
*Fagopyrum esculentum*	Blue LED	Rutin, orientin	Antioxidation	Nam et al. [68]
*Hypericum perforatum*	Red LED	Hypericin	Bioactive compound production	Sobhani Najafabadi et al. [69]
*Oryza sativa* cv. Dongjin	Red LED	*PPO1*	Gene expression	Tran and Jung [70]
*Cordyceps militaris*	Red and Blue (1:1) LED	Cordycepin	Bioactive compound production	Chiang et al. [71]
*Agastache rugosa*	White LED	Rosmarinic acid, *C4H, TAT, CHI*	Bioactive compound production, gene expression	Park et al. [72]
*Ocimum basilicum* purple varieties ‘Ardestan’	Red LED	α-pinene	Bioactive compound production	Hosseini et al. [73]
*Artemisia annua* L.	Blue LED	*ADS*, artemisinin	Bioactive compound production, gene expression	Lopes et al. [74]
*Lactuca sativa* ‘Sunmang’	Red, Blue and Far red (2:8:1.4) LED	Chlorogenic acid	Antioxidation	Lee et al. [75]
*Mesembryanthemum crystallinum* L.	Red and Blue (1:9) LED	Myo-inositiol, pinitol	Bioactive compound production	Kim et al. [76]
*Polygonum tinctorium* cv. senbon	Blue LED	*IGS, BGL*	Gene expression	Nakai et al. [77]
*Paecilomyces japonica*	Red:Blue (3:7) LED	Cordycepin	Bioactive compound production	Ha et al. [78]
*Carpesium triste* Maxim	Red and Blue (1:1) LED	CAT, POD, SOD and APX	Enzyme activity	Zhao et al. [42]
*Cnidium officinale*	Blue and White LED	CAT	Enzyme activity	Adila et al. [46]
Wheat	Blue-light	CAT	Enzyme activity	Causin et al. [43]
Rye	Blue-light	CAT	Enzyme activity	Schmidt et al. [47]
*Prunus avium,* Strawberry	Blue-LED	SOD	Enzyme activity	Franck et al. [50], Tian et al. [79]
*Gladiolus hybridus*	Blue LED	SOD and CAT	Enzyme activity	Gupta Dutta and Datta [80]
*Albizia adorratissima*	Blue LED	SOD and CAT	Enzyme activity	Rajeswari and Paliwal [52]
Barley	Red-LED	ɣ-tocopherol	Bioactive compound production	Koga et al. [81]
Apple	Yellow-LED	Tocopherol	Bioactive compound production	Kokaji et al. [82]
Basil	Red-LED	α-tocopherol	Bioactive compound production	Samuoliene et al. [53]
Beet and parsley	Blue-LED	Tocopherol	Bioactive compound production	Samuoliene et al. [83]
Pea	Red-LED	b-caretene	Bioactive compound production	Wu et al. [84]
Citrus fruit	Red-LED	b-cryptoxanthin	Bioactive compound production	Ma et al. [85]
Tomato	Red-LED	Lycopene	Bioactive compound production	Liu et al. [86]
Buckwheat	White-LED	Carotenoid	Bioactive compound production	Tuan et al. [87]
Citrus	Blue-LED	*CitPSY, CitZDS, CitPDS, CitLCY,*	Gene expression	Zhang et al. [88]
Broccoli	Short duration of Blue-LED	BC and VIO	Bioactive compound production	Kopsell and Sams [89]
Grape	Blue-LED	Anthocyanin	Bioactive compound production	Rodyoung et al. [90]
Buckwheat	Blue-LED	Anthocyanin	Bioactive compound production	Thwe et al. [91]
Wheat sprout	Blue-LED	*p*-coumaric acid, epicatechin	Bioactive compound production	Cuong et al. [49],
Lettuce	Red-LED	Anthocyanin	Bioactive compound production	Li and Kubota [92]; Stutte et al. [93]
Mustard	Red-LED	Anthocyanin	Bioactive compound production	Brazaityte et al. [94]
Cabbage	Red-LED	Anthocyanin	Bioactive compound production	Qian et al. [95]
Apples	Red-LED	Anthocyanin	Bioactive compound production	Lekkham et al. [96]
Grape	Blue LED and Red LED	*MYB* transcription factor genes	Gene expression	Koes et al. [97]
Grape	Blue-LED	*V1MYBA1-2, VIMYBA2* and *VvUFGT* increased	Gene expression	Rodyoung et al. [90]
Basil	RED-LED	TPC	Bioactive compound production	Samueliene et al. [53]
Chinese kale sprouts	Blue-LED	TPC	Bioactive compound production	Qian et al. [95]
Chinese cabbage and lettuce	Blue-LED and Red LED	TPC	Bioactive compound production	Li et al. [98]
*Pachyrhizus erosus*	RED-LED	Malonyldaidzin, malonyl genistin, salicylic acid, *p*-hydrobenzoic acid and gentisic acid	Bioactive compound production	Chung et al. [4]
Wheat sprout	Blue-LED	*p*-coumaric acid, gallic acid, ferulic acid, hydroxybenzoic acid	Bioactive compound production	Park et al. [99]
Wheat sprout	Blue-LED	*TaPA1,2, TaC4H, TaHCI, 1, TaCHS* and *TaF3H genes*	Gene expression	Cuong et al. [49]
*Cyclocarya paliurus*	Blue-LED.	(kaempferol, isoquercitrin and quercetin Phenylalanine ammonia lyase, PAL; 4-coumaroyl CoA-ligase, 4CL; and chalcone synthase, CHS	Bioactive compound production gene expression	Liu et al. [100]

## Data Availability

Not applicable.
